# Endovascular management of splenic artery aneurysms in women of childbearing age: a case report and a review of literature

**DOI:** 10.3389/fsurg.2025.1672059

**Published:** 2025-10-21

**Authors:** Karim Kanbar, Nadim Muallem, Jamal J. Hoballah

**Affiliations:** 1 Faculty of Medicine, American University of Beirut, Beirut, Lebanon; 2 Department of Diagnostic Radiology, American University of Beirut Medical Center, Beirut, Lebanon; 3 Division of Vascular Surgery, Department of Surgery, American University of Beirut Medical Center, Beirut, Lebanon

**Keywords:** splenic artery aneurysm, endovascular repair, vascular complications, pregnancy risk, arterial stenting, splenic perfusion, aneurysm exclusion

## Abstract

**Introduction:**

Splenic artery aneurysms (SAAs), though rare, are the most common splanchnic artery aneurysms and carry high maternal and fetal mortality if ruptured during pregnancy. Despite growing awareness, optimal long-term management and follow-up in women of childbearing age remain unclear.

**Presentation of case:**

We report a case of a healthy 37-year-old gravida 2 para 2 woman with an incidentally discovered 1.4 cm mid-splenic artery aneurysm on follow-up imaging after gastric banding. Due to her reproductive potential, endovascular treatment was performed. Coil embolization of two small branches arising from the aneurysm sac was followed by placement of a balloon-mounted 7 mm covered stent across the aneurysm neck. The procedure was uncomplicated, and post-intervention imaging confirmed aneurysm exclusion and preserved distal flow. Follow-up at 3 and 6 months showed complete thrombosis of the sac, patent stent, and no evidence of infarction. Ten months later, the patient had an uneventful pregnancy and delivery.

**Discussion:**

Endovascular approaches are increasingly preferred over surgery due to lower morbidity and better fertility preservation. Coiling is effective but may risk recanalization or infarction. Covered stents offer structural stability, preserve splenic flow, and allow clearer imaging follow-up. However, challenges include delivery in tortuous vessels and a potential endoleak. In our case, combining coiling of branches with stenting ensured aneurysm exclusion while supporting safe pregnancy.

**Conclusion:**

Stent grafting with selective coiling is a safe and effective option for managing SAAs in women of childbearing age. This case demonstrates successful treatment with long-term follow-up through pregnancy, though further research is needed to establish definitive guidelines.

## 1 Introduction

While being the most common splanchnic artery aneurysm and the third most common type of abdominal aneurysm (1, 2) splenic artery aneurysms (SAA) remain, nevertheless rare and fatal, with prevalence ranging from 0.2% to 10.4% in the general population and less than 0.1% in women of childbearing age (3–5). A recent large retrospective study has shown that 79% of cases of SAA were women (6) who are 4 times more likely to be affected (4). The true prevalence of SAA, however, is unknown due to most cases being discovered incidentally or after the emergence of symptoms during ultrasound imaging in pregnancy (7). Nearly 95% of SAA ruptures occur during pregnancy or labor (8), most commonly in the third trimester (9). Thus, SAAs entail a significant danger to pregnant patients, as their rupture leads to maternal mortality rates of up to 75% and fetal mortality rates as high as 90% (9).

A previously healthy 37-year-old gravida 2 para 2 previously healthy woman was found to have an SAA in the middle third of the splenic artery of 1.4 cm diameter on CTA, discovered on follow-up post gastric banding. The physical examination was normal, and laboratory results and vital signs were also normal. The woman's past medical and family history was unremarkable. The patient had a surgical history of a cesarean section 4 years prior and laparoscopic gastric banding. Due to the patient being of child-bearing age, intervention was recommended, and the patient was deemed a candidate for an endovascular approach. Table 1 summarizes the clinical course of this episode of care.

**Table 1:** Timeline of patient's episode of care, including diagnosis, intervention, follow-up, pregnancy, and postpartum outcomes.

Time point	Event/Intervention	Findings/Outcome
Patient characteristics	37-year-old G2P2 woman; incidental type II 1.4 cm SAA on CTA (post–gastric banding follow-up)	Normal exam, vitals, and labs; past history: C-section, gastric banding
Decision-making	Patient is of childbearing age thus intervention was recommended	Candidate for endovascular approach
Procedure (Day 0)	Right femoral artery access (modified Seldinger), coil embolization of 2 minor branches, and covered stent placement across aneurysm neck	Successful exclusion of aneurysm sac, preserved flow in dominant branch, no intra/post-op complications
Post-op	Started dual antiplatelet therapy (aspirin 81 mg + clopidogrel 75 mg daily)	Stable
3-month follow-up	CT angiography	Stent well-positioned, no hematoma or leak, thrombosed/resolved sac, spleen well-perfused
6-month follow-up	CT angiography	Findings unchanged; no infarction, no reintervention needed
10 months	Prenatal evaluation	No contraindications to pregnancy
12 months	Pregnancy achieved	Routine prenatal cared; dual antiplatelet therapy stopped at 12 weeks due to bleeding
34 weeks gestation	Delivery	Live birth; no maternal or neonatal long-term complications
Postpartum	Resumed dual antiplatelet therapy	Patient remained asymptomatic

SAA, splenic artery aneurysm; CTA, computed tomography angiography; G2P2, gravida 2, para 2.

Due to an increased predisposition to rupture and subsequent high mortality in pregnancy, early diagnosis and management of SAA in the childbearing population is crucial to avoid the loss of two lives. However, the preferred treatment and long-term follow-up approaches in patients in the childbearing age remain unclear. Herein, we present a case of a woman of childbearing age who underwent successful stenting of splenic artery aneurysm and coil embolization of its feeding branches, which was followed by an uncomplicated childbirth. Additionally, we review the current literature surrounding the endovascular management of SAAs in women of childbearing age.

## 2 Material and methods

The patient was incidentally found to have a 1.4 cm splenic artery aneurysm on CTA during post-gastric banding follow-up, and its size, location, and branch anatomy guided management decisions. Diagnostic challenges included the aneurysm being asymptomatic and distinguishing it from visceral artery pseudoaneurysms, segmental arterial mediolysis, or other visceral artery dilatations. Differential diagnosis considered pseudoaneurysm, arterial ectasia, and other visceral artery aneurysms, while prognostic considerations focused on rupture risk, especially during pregnancy which necessitate endovascular management.

**Figure 1:**
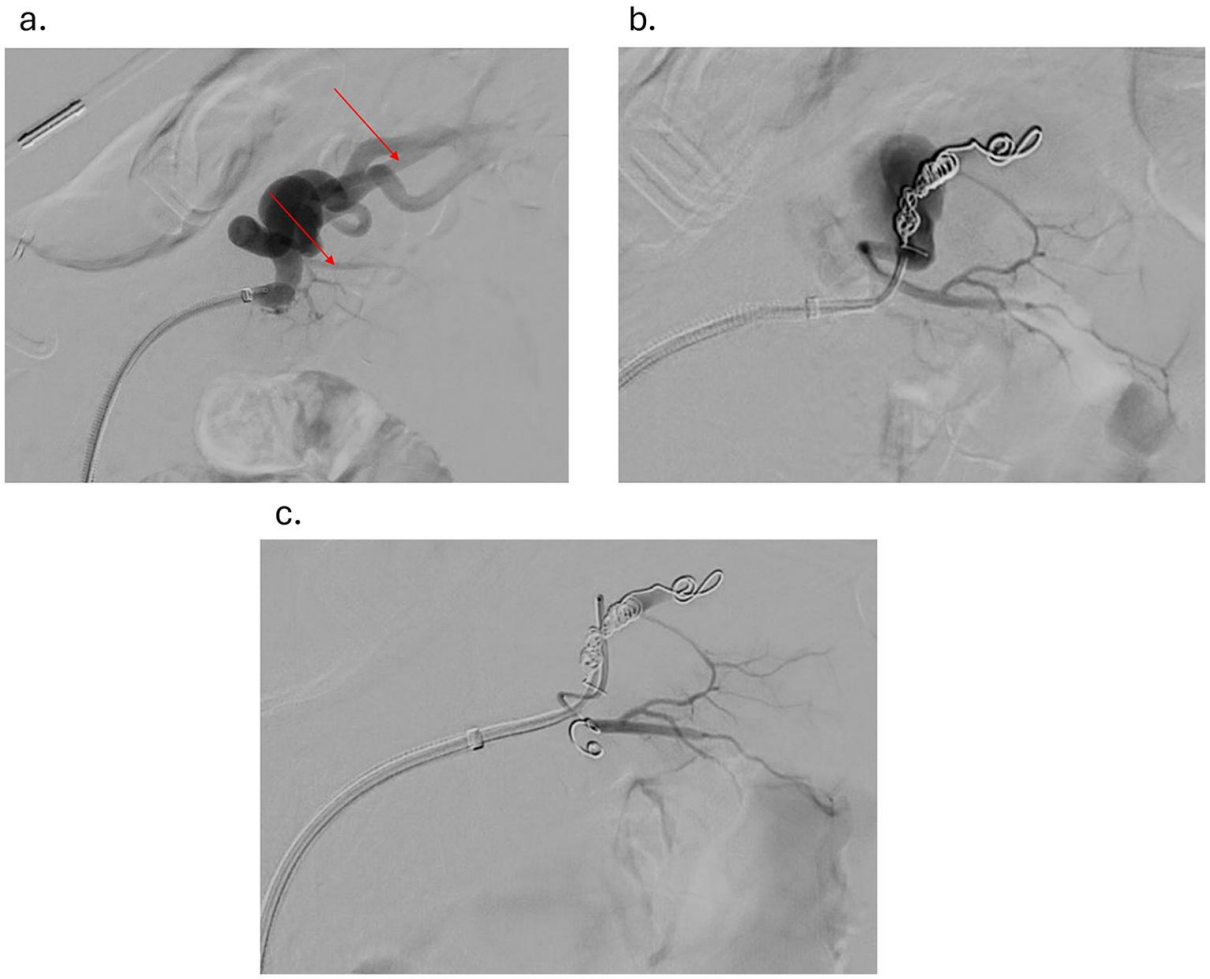
**(a)** angiogram showing splenic aneurysm with two branches originating from the aneurysm (red arrows) **(b)** angiogram showing splenic aneurysm after coiling of the first branch originating from the aneurysm **(c)** angiogram showing splenic aneurysm after coiling of the second branch originating from the aneurysm.

**Figure 2:**
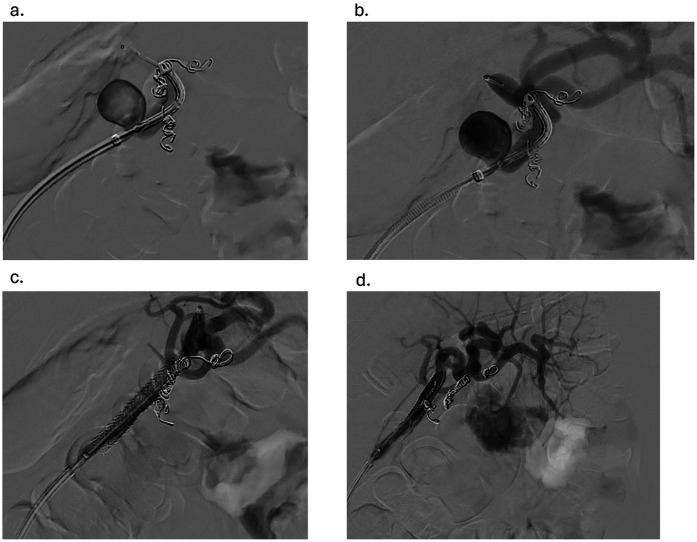
**(a)** advancement of the balloon-mounted 7 mm covered stent across the aneurysm **(b)** confirmation of the desired location of the endoprosthesis with sequential arteriography **(c)** withdrawal of the flexor sheath to the desired location and the stent graft deployed **(d)** angiogram showing the aneurysm post-coiling of branches and stent graft placement.

Under sterile conditions, the right femoral artery was accessed using the modified Seldinger technique, and a 5 French vascular sheath was inserted. The splenic artery was selected using a 5-French Cobra catheter and a 0.035-inch hydrophilic Glidewire. A contrast run at the proximal splenic artery showed a large splenic artery aneurysm arising at the bifurcation of major branches. The vascular sheath was exchanged for a larger flexor sheath, which was advanced into the distal splenic artery just proximal to the splenic artery aneurysm. Two minor branches were identified as arising from the aneurysmal sac (Figure 1a). These were individually selected with a microcatheter and embolized with seven Cook Medical® Micronester® embolization coils to prevent backfilling of the aneurysm(Figures 1b,c). Following coil embolization, the remaining dominant branch was selected, and the flexor sheath was advanced across the aneurysm neck, confirming adequate exclusion and distal flow. Then a single balloon-mounted 7 mm Bentley® BeGraft® covered stent was advanced and deployed across the aneurysm neck and unsheathed by slowly retracting the flexor sheath and confirming the appropriate desired location with sequential arteriography (Figures 2a–c). Post-stent deployment angiography showed complete exclusion of the aneurysmal sac with preserved patent flow into the dominant splenic artery branch (Figure 2d). There were no intraoperative and acute postoperative complications. The patient was placed on dual antiplatelet therapy composed of 81 mg aspirin and 75 mg clopidogrel per day. In brief, successful isolation of a large proximal splenic artery aneurysm and preservation of patency of the dominant splenic artery branch were performed by using a covered stent and coiling of minor branches to prevent aneurysm backfilling.

## 3 Results

**Figure 3:**
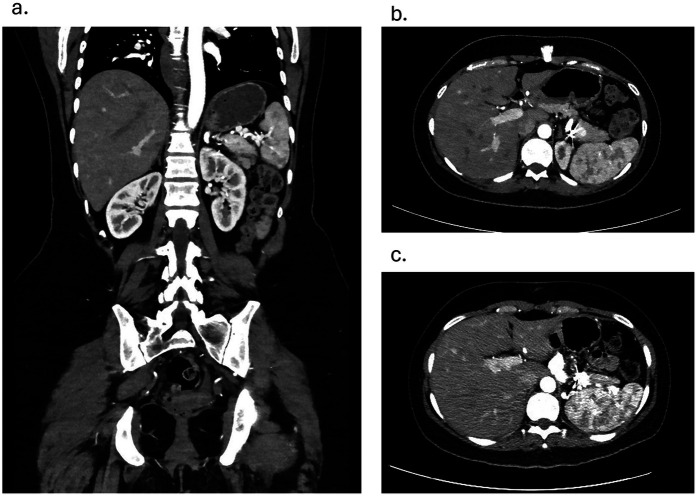
**(a)** coronal view contrast enhanced CT angiography showing patent stent and a well-perfused spleen on 3 months follow-up. **(b)** Axial view Contrast Enhanced CT angiography showing coiled branches and a well-perfused spleen on 3 months follow-up **c.** Axial view Contrast Enhanced CT angiography showing coiled branches and a well-perfused spleen on 6 months follow-up.

Follow-up after 3 and 6 months on CT angiography revealed that the stent was in an adequate position with no hematoma or contrast leakage (Figures 3a–c). Thrombosis of the aneurysm sac was confirmed as the excluded sac was not visualized and likely has resolved. The spleen was well-perfused and normal in size, and no infarction was noted. No reintervention was needed or performed, and no enlargement of the distal SAA was seen. The patient remained asymptomatic throughout. Prenatal evaluation 10 months later revealed no issues preventing pregnancy, and the patient became pregnant a year after the procedure, delivering at 34 weeks without long-term complications. The child remains healthy with normal growth. Throughout pregnancy the patient received routine prenatal care, and no additional imaging was performed to assess the repair site. During pregnancy, the patient remained on the same dual antiplatelet therapy with unchanged dosage and frequency; however, it was discontinued at 12 weeks due to bleeding, and then resumed postpartum at the same regimen. From the patient's perspective, she was reassured by the minimally invasive treatment that preserved her fertility and enabled a safe pregnancy and healthy child.

## 4 Discussion

A splenic artery is considered aneurysmal when its diameter exhibits a focal dilation that exceeds 50% of the normal vessel diameter, which ranges from 0.43 cm to 0.49 cm (5). The risk of rupture of a true aneurysm increases as it becomes >2 cm in diameter (10) while the mean size of SAA during pregnancy was found to be 23.0 ± 13.6 mm (11). SAAs can be classified into several types to facilitate treatment (12). For example, Type I are saccular aneurysms with a narrow neck, Type II are wide-necked saccular or fusiform aneurysms located in the main trunk as in our case, Type III are true SAAs in the distal part of the artery, and Type IVa are ostial lesions, or those sharing a common trunk with the celiac artery and superior mesenteric artery (SMA) (12).

Clinically, the high mortality of SAAs in pregnant patients is primarily due to their asymptomatic presentation, the rapid clinical deterioration after rupture, and their incorrect diagnosis since they can mimic other obstetric emergencies such as placental abruption (9, 13, 14). Estrogen and progesterone may compromise medial integrity (15, 16) which, when combined with increased elasticity of the artery mediated by relaxin (9, 17) results in aneurysmal dilatation and potential rupture. Apart from hormonal changes, increased physiological demands and changes during pregnancy, including increased cardiac output, increased blood volume, portal hypertension, and elevated mechanical pressure by the growing gravid uterus in late pregnancy, are other contributing factors (5, 18). Moreover, multiparous women and those with portal hypertension have an increased risk of rupture due to higher portal blood flow (5, 19, 20).

Rupture during pregnancy commonly presents as severe, sudden, sharp left upper quadrant abdominal pain radiating to the tip of the shoulder (Kehr's sign) with signs of hypovolemic shock (21). This describes a sudden stage rupture; however, two-stage ruptures may also occur in nearly a quarter of cases whereby the initial rupture is contained within the lesser sac by omentum and/or blood clots blocking the foramen of Winslow while the patient remains asymptomatic. After 6–96 h, free rupture occurs into the greater sac and subsequently presents with symptoms of sudden initial pain followed by circulatory collapse due to shock (22). Thus, diagnosis and prompt treatment in the window after primary rupture is critical for the survival of the patient and fetus, even if the patient appears stable (22).

Digital subtraction angiography is considered a gold standard in SAA diagnosis; however, due to its risks during pregnancy, it is usually performed only with concomitant radiological intervention such as when coil embolization or endoluminal stenting is required (9). Due to their noninvasiveness and safety, Ultrasound and Doppler Ultrasound are preferred during pregnancy, revealing aneurysmal flow dynamics but operator dependence, patient factors, and the risk of underestimating lesion size with color Doppler limit their use (13). Lastly, while abdominal x-ray may be useful for revealing calcified SAAs, and CT or MRI can be employed for 3d visualization of the splenic artery, both are avoided due to pregnancy-associated risks (13).

The latest clinical practice guidelines from the Society of Vascular Surgery recommend monitoring over repair of small, asymptomatic aneurysms that are less than 3 cm, which show little or no growth in patients with low risk of rupture (23). Those that are larger than 3 cm showing signs of growth or symptoms are recommended to be treated in patients with a low risk of rupture (23). On the other hand, in women of childbearing age, true aneurysms and pseudoaneurysms of any size are recommended to be treated due to the aforementioned risk of rupture (23). If anatomically feasible or otherwise contraindicated, endoscopic repair of unruptured SAAs in such populations is recommended (23).

Open surgical approaches are generally complicated with mortality of 1.3% and morbidity is 9% due to their highly invasive nature, often requiring the resection of the spleen, pancreas, or other adjacent organs (24). The management of anatomically suitable SAA endovascular techniques confers several advantages over open surgical techniques, including reduced invasiveness, shortened hospital stay, improved patient quality of life peri-operatively, decreased morbidity rates compared to open repair, decreased 30-day mortality rates, and decreased cost across most population groups (25–32). Furthermore, endovascular management is preferable in the presence of challenging surgical conditions like pancreatitis, sepsis, or multiple prior abdominal surgeries (33). In a childbearing age, by avoiding large surgical scars and reducing the potential for complications in subsequent pregnancies, endovascular management offers a safer and more fertility-preserving option compared to open surgery. Success rates of various endovascular procedures were shown to reach up to 100% in several studies (29, 34–36). The average length of hospital stay was found to be 5.5 ± 3.2 days in one study with no significant difference among different endovascular approaches (34). Complications common to all endovascular procedures include vessel rupture, dissection, and occlusion (37).

Embolization methods in the treatment of SAA usually involve coiling. Anatomical features of the splenic artery make transcatheter coiling a more feasible approach in certain situations, whereby the maneuverability of coils through the long and tortuous nature of the splenic artery makes them a desirable choice for the treatment of Type III SAAs (12, 34). Also, endovascular aneurysmal coiling is the preferred treatment method for managing Type I SAAs and complex Type IVa SAAs (5, 12, 38). Postoperative complications which may be poorly tolerated during pregnancy or pose significant prenatal risk are a major concern with this technique. Postembolization syndrome, characterized by pain, fever, and other systemic symptoms, can develop in up to 20%–25% of patients (34, 39). However, postembolization syndrome is reversible. Splenic infarction due to distal embolization, an early complication of this procedure, is another limitation of this approach, particularly in the treatment of giant aneurysms and in wide-necked aneurysms (33, 40, 41). Portal hypertension has been described as a major risk factor for splenic infarction following transcatheter embolization, occurring in more than half of patients with this condition (39). High aneurysm recanalization rates, a late complication of this procedure occur up to 12.5%, due to the frequent occurrence of incomplete embolization or reperfusion of the aneurysmal sac through collateral vessels, resulting in increased rates of reintervention. However, several techniques such as the use of n-Butyl Cyanoacrylate glue (42, 43) and low-profile microembolization platforms (44) have shown potential to overcome this issue. Double microcatheter techniques utilizing single access have been used successfully in the management of SAAs with anomalous anatomy (45) and have shown potential in the treatment of wide-necked SAAs with a low risk of distal organ infarction (38).

Stent-graft positioning (covered stent) is a viable alternative, especially where coiling is difficult, such as Type II SAAs (5, 12). It is important to have both proximal and distal landing zones that are large enough (10–15 mm) and adequate preoperative size matching for technical success, which can further be increased if the aneurysm occurs in the middle or proximal third of a vessel lacking marked tortuosity (40, 46). This decreases the risk of splenic ischemia (including that caused by distal embolization following coiling), preservation of splenic arterial flow (37, 46, 47). Stent grafting facilitates imaging follow-up by eliminating the artifact typically caused by coil embolization, thereby providing clearer and more accurate images for ongoing assessment and monitoring of the aneurysm's condition (46). Stent migration, a significant complication of this procedure, has been found to occur in 7.3% of cases and could be caused by unsuitable oversizing at the preoperative planning stage, which is common when treating urgent and severe situations (37). A major complication specific to stenting grafting is difficult delivery due to vessel tortuosity. The stiff nature of the guidewire system complicates access, making it not feasible in markedly tortuous splenic arteries, as the risk of failed delivery is high (46). Likewise, delivery into the distal third of the splenic artery or a narrow splenic artery is difficult (40). It is important to note that splenic infarction may still occur as a late complication of stenting due to partial or complete stent thrombosis; however, this can be less clinically relevant or asymptomatic in some cases and can be swiftly managed with conservative treatment (36, 48). In a true SAA with branching vessels, there is a risk of developing a low-pressure leak similar to a type II endoleak seen in aortic stent grafting (40). Consequently, if thorough preprocedural assessment of the aneurysm finds branching vessels, it may be necessary to embolize these vessels before stent graft placement (40). In our case, we followed this approach even though the splenic artery was not aberrant, ensuring the successful isolation of the aneurysm and preventing a type II endoleak related to collateral blood flow that can affect a potential pregnancy that our patient was planning for.

Other endovascular techniques have shown success in the management of SAAs. Flow diverter stents, also known as multilayered stents, originally employed in the management of intracranial aneurysms, are one novel alternative. They facilitate the reconstruction of the parent vessel by causing the blood flow in the aneurysm to become stagnant with subsequent thrombosis and the formation of a new endothelial layer over the aneurysm's ostium, effectively isolating it from the circulatory system (49). This approach can be used in tortuous and small caliber vessels that complicate the covered stent approach due to the increased flexibility and adaptability of flow diverter stents compared to conventional covered stents and when preservation of distal flow contraindicates both covered stents and transcatheter embolization (49). It was able to reduce the flow velocity within the aneurysm by up to 90%, in a multicenter retrospective study using the Cardiatis multilayer flow modulator, while enhancing laminar flow in the main artery and its side branches (50). The same study revealed that thrombosis of the aneurysm occurred in 93.3% of cases, with 86.9% and 90.7% primary and secondary patency, respectively, in all patients (50). Their flexibility additionally reduces the risk of vessel injury or rupture (49). In a recent multicenter study, treatment with the Derivo Peripheral Flow Diverter achieved complete aneurysm occlusion at 3 months and full stent patency at 12 months, with no major complications or mortality (32). Flow diverters are used off-label in this setting, requiring informed consent, and their high cost limits widespread use (49). Multiple overlapping bare stents resemble multilayer stents; however, they do not need to be designed before surgery, increasing their convenience and have shown a 75% clinical achievement rate and 100% rate of side branch patency in a preliminary small sample study (51).

Combination techniques serve as another alternative in aberrant or anatomically complex SAA arteries, such as those that are an ostial lesion of the splenic artery or originate from the superior mesenteric artery or Type IVa aneurysms (12). Combined stenting and embolization of aberrant SAAs, including SMA-origin cases, have shown long-term success with no adverse events during the perioperative period or follow-up (12, 52). The treatment of an SAA with a narrow entrance requiring maintained distal flow was achieved by using Viabahn short stent grafts and coiling of the draining arteries and demonstrated long-term success with a low postoperative risk of stent graft obstruction or stenosis (53). Apart from aberrant SAAs, the flexibility of a combination of bare stent-assisted coil embolization allows successful management of SAAs located at a branch of the major splenic artery or when stenting is difficult (47).

Although treatment according to the type of aneurysm is still being clarified and is recommended for SAAs of any size in the presence of symptoms, those who are growing, or those in a childbearing population, very little is known or reported on the long-term management and follow-up, especially in the case of the latter prior, during, and post-pregnancy. To the best of our knowledge, our case is the first case in which a patient in the childbearing population was followed during and post-pregnancy. Khoury et al. demonstrated increased in-hospital mortality among women of childbearing age undergoing inpatient SAA repair, particularly in nonelective settings and after splenectomy (54). Our case complements these findings by showing that elective, spleen-preserving endovascular repair not only achieves durable aneurysm exclusion and splenic preservation but can also be followed by an uncomplicated pregnancy and delivery. While Khoury's dataset included only one pregnant patient and could not assess pregnancy outcomes, our report extends their observations beyond in-hospital mortality to longer-term follow-up, thereby reinforcing the importance of elective repair in this high-risk population. Keeping in mind the potential long-term side effects that can arise with coiling, such as recanalization and incomplete embolization, and recognizing the added benefits of enhanced structural stability and better flow dynamics with stent use, which is especially important during the physiological changes of pregnancy, we have opted for the use of stent grafts. While much in the literature has been reported about using self-expanding stents, we have decided to use balloon angioplasty due to its better predictability, which is especially important with vessels that can increase in size, such as during pregnancy or aging. Furthermore, stent implantation combined with branch artery embolization instead of aneurysm lumen embolization plus branch artery embolization was chosen to preserve the patency of the splenic artery avoiding splenic infarction and its possible sequalae. In terms of monitoring, CT is a better choice; however, such repetitive exposure to ionizing radiation can increase the chances of cancer in the long term. In this regard, stent grafting could be more favorable for follow-up since it doesn't create an artifact similar to coiling, which is important in preparation for and during pregnancy.

## 5 Conclusion

SAAs, though uncommon, present substantial risks during pregnancy, leading to high maternal and fetal mortality rates if ruptured, especially in the third trimester. We report a healthy 37-year-old woman with an incidentally found 1.4 cm splenic artery aneurysm, successfully treated with coil embolization and a covered stent. Imaging confirmed aneurysm exclusion and preserved flow, followed by an uneventful pregnancy and delivery. Stent grafting was chosen and shown to be a good option due to its safety, structural stability, better flow dynamics, and easier follow-up via imaging, all of which are crucial considerations for long-term management to not only ensure effective treatment but also support ongoing monitoring and maintenance of vascular health over time, especially for the childbearing population. More research and long-term follow-up are needed to determine whether stent grafting or other interventions are safe and effective options for women of childbearing age.

## Data Availability

The original contributions presented in the study are included in the article/Supplementary Material, further inquiries can be directed to the corresponding authors.
